# Both the presence of a micropapillary component and the micropapillary predominant subtype predict poor prognosis after lung adenocarcinoma resection: a meta-analysis

**DOI:** 10.1186/s13019-020-01199-8

**Published:** 2020-06-29

**Authors:** Wei Wang, Zaoxiu Hu, Jie Zhao, Yunchao Huang, Sunyin Rao, Jichen Yang, Shouyong Xiao, Run Cao, Lianhua Ye

**Affiliations:** 1 Department of Thoracic Surgery, The Third Affiliated Hospital of Kunming Medical University, No. 519 Kunzhou Road, Xishan District, Kunming City, Yunnan Province China; 2 Department of Pathology, The Third Affiliated Hospital of Kunming Medical University, Kunming, China

**Keywords:** Presence of micropapillary component, Micropapillary predominant subtype, Lung adenocarcinoma, Prognosis, Meta-analysis

## Abstract

**Objective:**

It has been confirmed that the micropapillary (MP) pattern is a poor prognostic factor after resection of lung adenocarcinoma (ADC), but the proportion of the MP component as a prognostic criterion is still controversial. Hence, a meta-analysis was performed to evaluate whether the presence of an MP component has equal prognostic power as the MP predominant subtype.

**Methods:**

Literature retrieval was performed in the MEDLINE, EMBASE, and Cochrane databases until December 23, 2019. Eligible studies were selected based on the inclusion and exclusion criteria. The included studies were divided into two subgroups, the MP component subgroup and the MP predominant subgroup, according to the proportion of the MP pattern to analyse the effect of this pattern on disease-free survival (DFS) and overall survival (OS). The hazard ratio (HR) and 95% confidence interval (CI) were extracted from each study. Review Manager 5.3 was used for statistical analyses.

**Results:**

Finally, 10 studies, including a total of 4934 lung ADC patients, were included in this meta-analysis. Our results indicated a significantly worse pooled DFS (HR 1.62, 95% CI 1.20–2.21) and OS (HR 1.53, 95% CI 1.19–1.96) in the subgroup of MP predominant subtype patients. The pooled DFS (HR 1.80, 95% CI 1.45–2.85) and OS (HR 2.26, 95% CI 1.46–3.52) were also poor in the subgroup of patients with the presence of an MP component.

**Conclusions:**

Both the presence of an MP component and the MP predominant subtype are related to poor DFS and OS after lung ADC resection and represent adverse prognostic factor for lung ADC patients. However, there are some limitations in this meta-analysis, and quantitative stratification based on the proportion of the MP component is needed to explore its effect on prognosis of lung ADC patients in the future.

## Introduction

Primary bronchial lung cancer is one of the most malignant tumours with the highest morbidity and mortality in China and the world, seriously endangering human health [1]. Adenocarcinoma (ADC) is the most common histological type of lung cancer, accounting for almost 50% of all lung cancers [2]. The International Association for the Study of Lung Cancer, the American Thoracic Society and the European Respiratory Society (IASLC/ATS/ERS) proposed a new histological classification method for lung ADC in 2011 [3]. According to the recommendations of the new classification, the 5 main growth patterns (lepidic, acinar, papillary, micropapillary and solid) of invasive lung ADC should be recorded in 5% increments, and the pattern with the highest proportion determines the predominant subtype [4].

Studies have shown that the new classification is an independent predictor of disease-free survival (DFS) and overall survival (OS) in lung ADC [5–7]. In recent years, many studies have reported the poor prognosis of lung ADC with the micropapillary (MP)-predominant subtype [8, 9]. In addition, studies have shown that the MP predominant subtype of lung ADC is closely related to lymph node metastasis, vascular tumour thrombosis, visceral pleural invasion, and spread through air spaces (STAS) [10–12]. Two studies showed that the presence of an MP component does not suggest a poor prognosis for lung ADC [13, 14]. The proportion of the MP component as a prognostic criterion is still controversial. It is uncertain whether the presence of an MP component has equal prognostic power as the MP predominant subtype.

The aim of this study was to review the available data and conduct a meta-analysis, dividing the studies into two subgroups, the MP component subgroup and the MP predominant subgroup, according to the proportion of the MP pattern to clarify the effect of this pattern on the prognosis of lung ADC after resection.

## Methods

### Eligibility criteria

Eligible studies included observational studies assessing the significance of the lung ADC subtype for prognosis. The selected studies met the following criteria: (1) the research subjects were patients with confirmed lung ADC after surgical resection as evaluated by the comprehensive and detailed histological diagnostic model according to the new IASLC/ATS/ERS classification; (2) the MP pattern was defined as MP predominant or containing an MP component; (3) DFS and/or OS should be evaluated in the study; and (4) the hazard ratio (HR) of DFS and/or OS was obtained through multivariate Cox regression analysis. Studies meeting the following criteria were excluded: (1) editorials, letters, reviews, conference abstracts, and case reports; (2) studies published in a non-English language; (3) incomplete information or the HR and 95% CI could not be calculated; (4) the patients received neoadjuvant therapy before surgery; and (5) studies with un unclear definition of the MP pattern or inconsistent standards defining the presence of an MP component.

### Search strategy

The included studies were identified through an electronic search of the MEDLINE, EMBASE, and Cochrane databases, and the deadline was December 23, 2019. In addition, other related studies were obtained by scanning the reference lists of the included studies. Search terms were combined as follows: (micropapillary predominant OR micropapillary component OR micropapillary pattern OR micropapillary subtype OR micropapillary minor) AND (resect OR resection OR operate OR operation OR lobectomy OR limited resection) AND (cancer OR carcinoma OR tumour OR neoplasm) AND (lung OR pulmonary) AND (survival OR prognosis).

Two authors (WW and ZH) independently browsed the titles and abstracts of all the studies identified by the electronic searching and then acquired the full articles for all potentially relevant studies. Next, the full texts of these potentially relevant studies were read through by the previous two authors to select studies that met the inclusion criteria. In the censoring process, any inconsistencies between the two authors needed to be discussed with the senior author (LY).

### Data extraction and quality assessment

Data extraction was performed independently by the two authors (JZ and SR). The information needed to be extracted included: (1) first author, year of publication, study region, type of study, starting and ending years; (2) total number of patients, MP pattern definition, number of MP patients, Tumor Node Metastasis (TNM) staging distribution, surgical approach, and median follow-up time; and (3) the HR of DFS and/or OS and 95% CI.

The quality of included studies was evaluated independently by the two authors (JY and SX) after reading the full texts, and the Quality In Prognosis Studies (QUIPS) tool [15] was used to assess the risk of bias. Each domain was evaluated as having a low, medium or high bias risk from the six aspects including the research object, the study of lost follow-up, the measurement of prognostic factors, the measurement of outcomes, the confounding of research, and the statistical analysis of data and reporting. Any inconsistencies between the two authors were discussed with the senior author (YH).

### Data analysis

The Review Manager 5.3 software (Rev-Man, version 5.3, Copenhagen: Nordic Cochrane Centre, Cochrane Collaboration, 2014) was used in this study for meta-analysis of survival data. According to the HR and 95% CI reported in the included studies, the Ln (HR) and the standard error (SE) were calculated and analyzed by using the generic inverse variance. The pooled HR of DFS or OS was chosen as the effect index. The included studies were divided into two subgroups, the MP component subgroup and the MP predominant subgroup, according to the proportion of the MP pattern. The effect of the MP pattern on DFS and OS in patients with lung ADC was analysed. The heterogeneity between the included studies was assessed using the Cochrane Q test and I^2^ value [16]. If P>0.1 or I^2^<50%, the heterogeneity was considered to be non-significant, and the fixed effects model was used; otherwise, the random effects model was used [16]. Begg’s funnel plot and Egger’s linear regression test were used to assess the publishing bias. All *P* values were two-sided, and *P* < 0.05 was considered statistically significant.

## Results

### Study characteristics

**Fig. 1:**
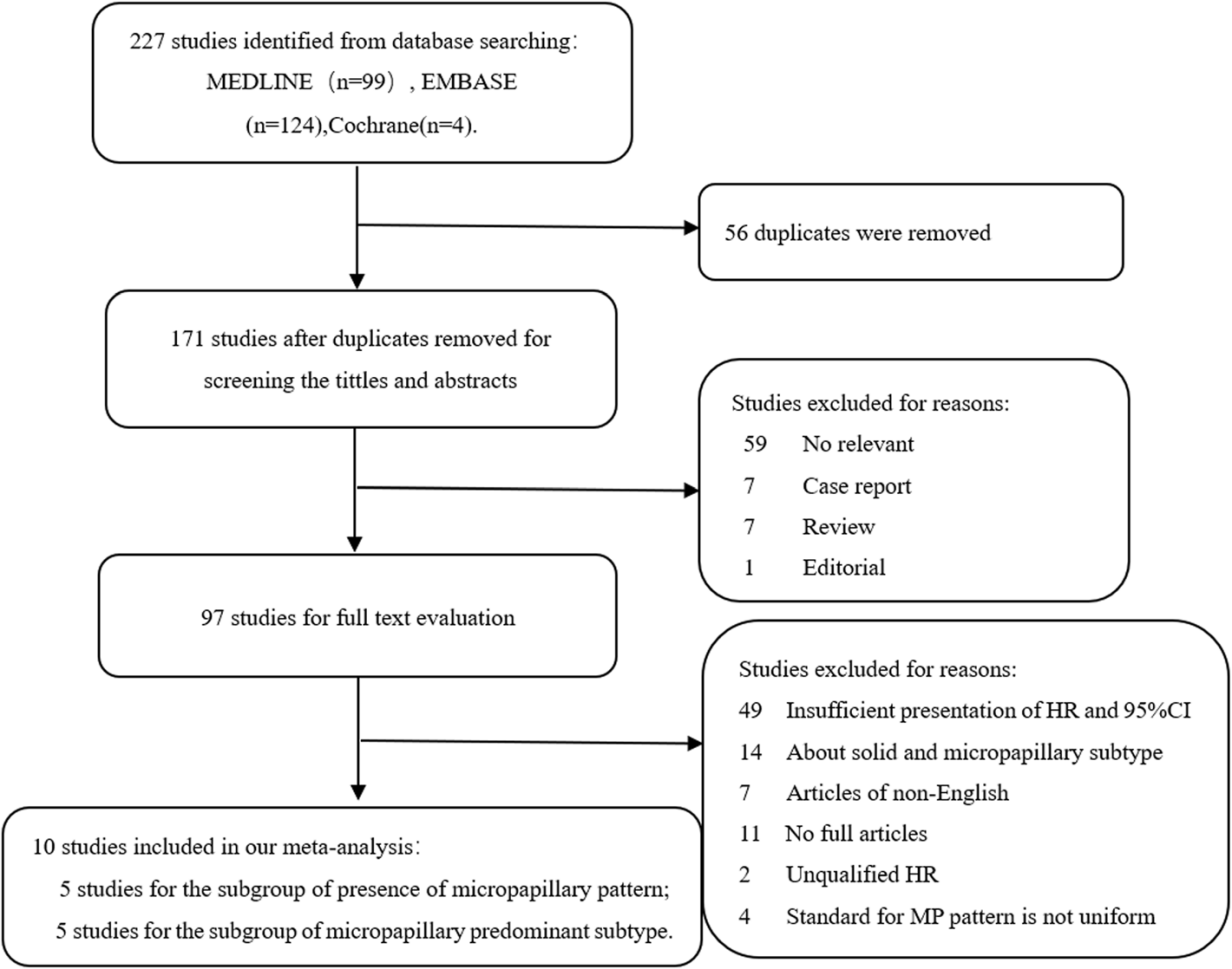
Flow chart of the identification of included studies

A total of 227 potentially relevant studies were retrieved from the MEDLINE, EMBASE, and Cochrane databases. The screening process for available studies is shown in Fig. 1. Finally, 10 studies published from 2014 to 2018,which included a total of 4934 patients, were included in this meta-analysis. All included studies were retrospective; 2 of them came from Japan, 2 from South Korea, 4 from China, 1 from Germany and 1 from Australia. Five studies described the effect of the presence of an MP component on prognosis, and 5 studies described the effects of the MP predominant subtype on prognosis. DFS was used as a single prognostic indicator in 5 studies, 2 studies described both DFS and OS, and the remaining 3 studies only described OS. The HR and 95% CI in each study were obtained through multivariate Cox regression analysis, and then the pooled HR of the meta-analysis was calculated. The characteristics of the included studies are summarized in Table 1. The quality evaluation results of the included studies are shown in Table S1.

**Table 1:** The characteristics of the included studies

First Author (year)	Study Region	Starting and Ending Year	MP Pattern Definition	Total Number of Patients	Number of MP Patients	Age	TNM Staging Distribution				Surgical Approach				Median Follow-up Time	Outcome
							I	II	III	IV	Lobecto-my	Limited Resection	Pneumon-ectomy	Bilob- ectomy		
**Presence of micropapillary component**																
Liu (2014) [17]	China	2007.5–2012.2	MP component	248	79	60 (24–81)	110	30	81	27						OS
Tsubokawa(2016) [18]	Japan	2006.4–2010.12	MP component	347	49	unclear	325	11	11		192	155			40.6	DFS
Moon (2016) [19]	Korea	2010.1–2014.12	MP component	168	37	unclear	133	35			163			3	29.1 (0.4–62.2)	DFS
Yao (2016) [13]	China	2012.9–2014.2	MP component	20	5	62 (43–80)	7	8	5		17	3			30 (24–41)	DFS
Yi (2018) [14]	Korea	2009–2012	MP component	368	141	62.6 (30–91)	368				250	114	1		43 (0–73.9)	DFS
**Micropapillary predominant subtype**																
Westaway (2013) [20]	Australia	2000–2010	MP predominant	152	8	68 (31–100)	79	42	31		133	8	11			OS
Sun (2014) [21]	China	2002.1–2011.12	MP predominant	136	22	57.6 (34–79)	136				119			17	74 (21–145)	DFS, OS
Warth (2015) [22]	Germany	2002–2010	MP predominant	674	53	62.6	264	145	243	22	549	22	87	16	38.2	DFS
Watanabe (2015) [8]	Japan	1998–2007	MP predominant	2316	139	unclear	I + II 1912		III + IV 404							OS
Zhang(2016) [9]	China	2007.1–2010.6	MP predominant	505	49	57 (24–83)	221	81	203		443	19	11	32	43 (22.9–57.5)	DFS, OS

MP pattern definition: Semiquantitative record various patterns that may be present in 5% increments, the presence of MP component is defined as the proportion of MP pattern > 5%, and the MP predominant subtype defined as the MP pattern present in the largest proportion [3, 4]

### Effects of the MP pattern on DFS after lung ADC resection

**Fig. 2:**
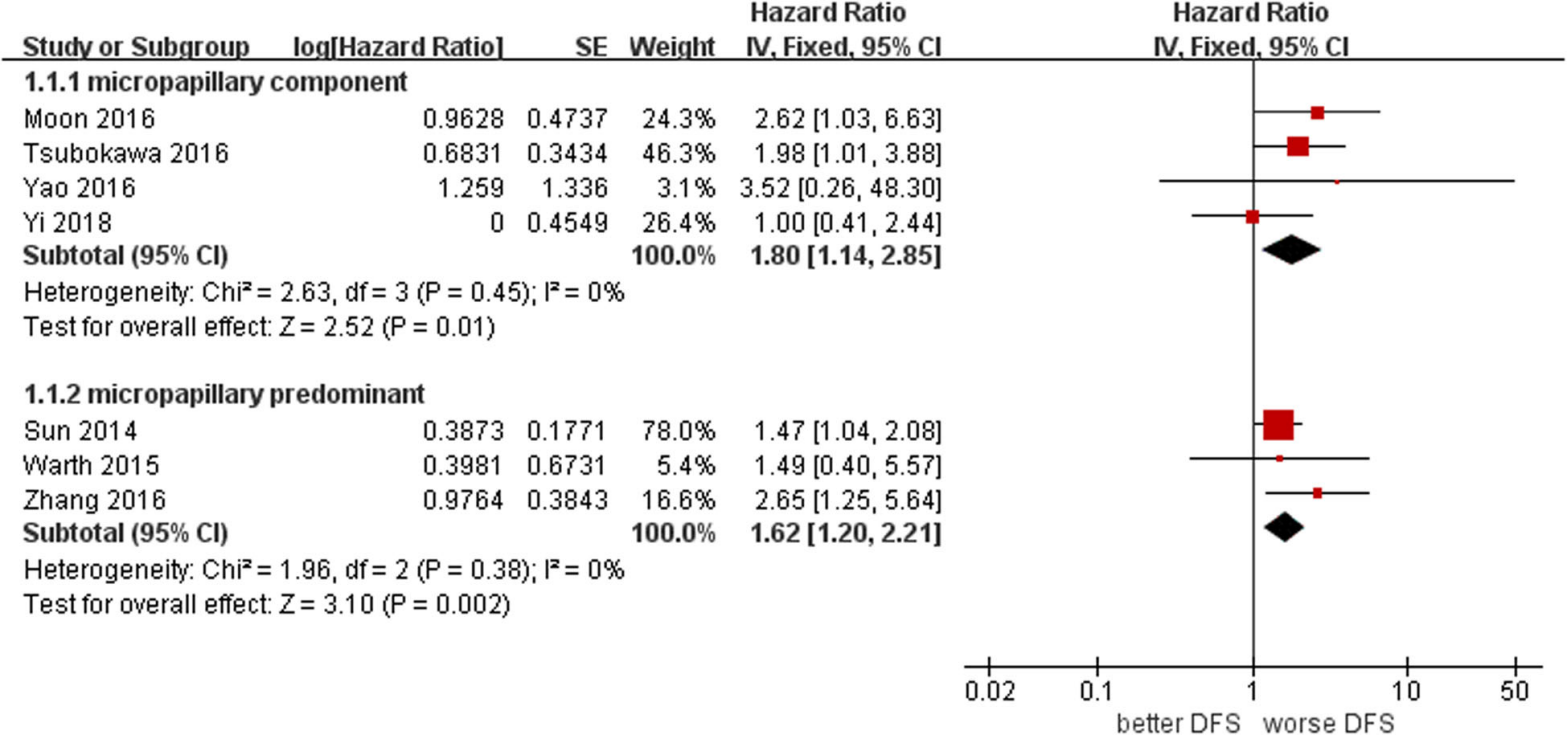
Forest plot of pooled HR for DFS among the included studies

Four of the five studies in the subgroup with the presence of an MP component reported the HR of DFS obtained by multivariate Cox analysis from 903 patients. The fixed effects meta-analysis showed that the DFS of MP component-positive patients was significantly worse than that of MP component-negative patients (HR 1.80, 95% CI 1.14–2.85, I^2^ = 0%, *P* = 0.01) (Fig. 2).

Three of the five studies in the subgroup of the MP predominant subtype reported the HR of DFS obtained by multivariate Cox analysis from 1315 patients. The fixed effects meta-analysis showed that the DFS of MP predominant subtype patients was also significantly worse than that of MP nondominant subtype patients (HR 1.62, 95% CI 1.20–2.21, I^2^ = 0%, *P* = 0.002) (Fig. 2).

### Effects of the MP pattern on OS after lung ADC resection

**Fig. 3:**
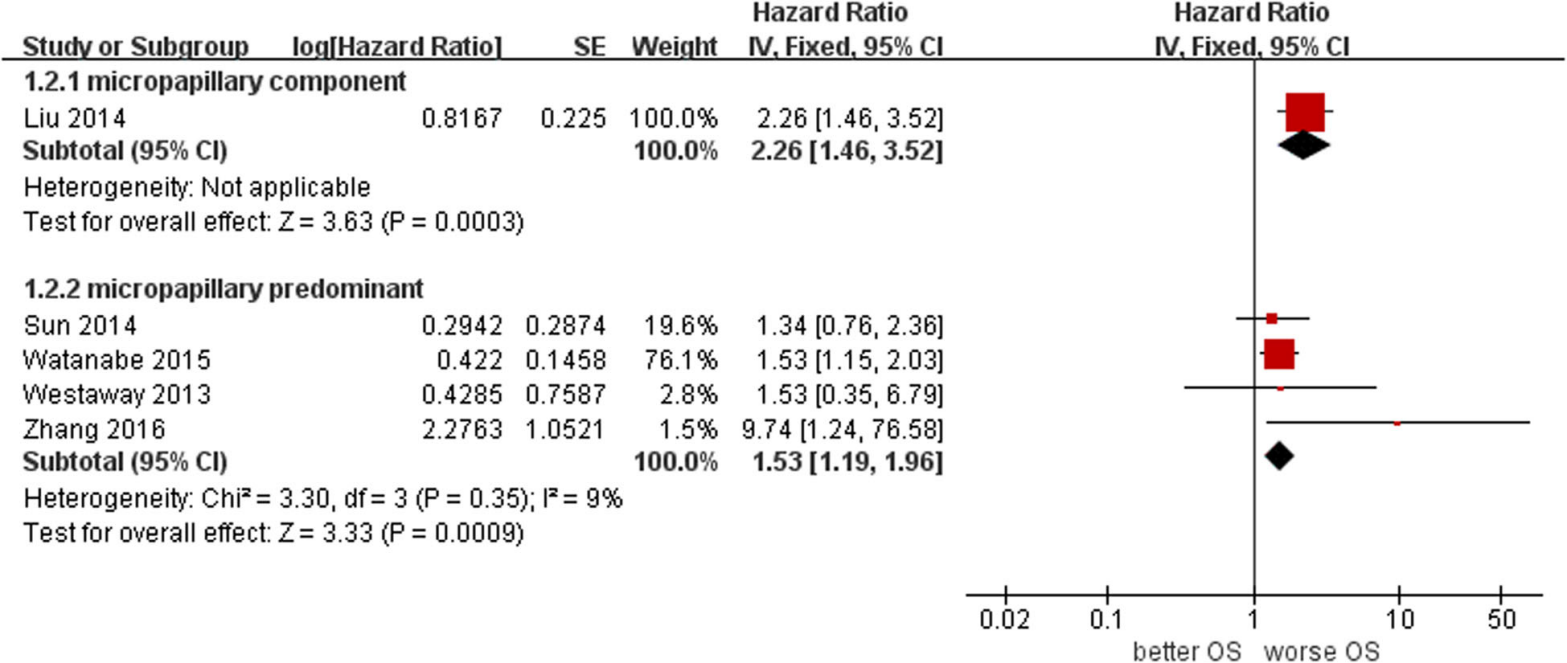
Forest plot of pooled HR for OS among the included studies

One of the five studies in the subgroup with the presence of an MP component reported the HR of OS observed from 248 patients. The OS of MP component-positive patients was significantly worse than that of MP component-negative patients (HR 2.26, 95% CI 1.46–3.52, *P* = 0.0003) (Fig. 3).

Four of the five studies in the subgroup of the MP predominant subtype reported the HR of OS obtained by multivariate Cox analysis from 3109 patients. The fixed effects meta-analysis showed that the OS of MP predominant subtype patients was also significantly worse than that of MP nondominant subtype patients (HR 1.53, 95% CI 1.19–1.96, I^2^ = 9%, *P* = 0.0009) (Fig. 3).

### Publication bias

Publication bias was evaluated by Begg’s funnel plot and Egger’s linear regression test. There was no publication bias among the 7 studies describing DFS (*P* = 0.379>0.05). No significant publication bias was found among the 5 studies describing OS (*P* = 0.390 > 0.05).

## Discussion

Our meta-analysis of 10 studies (including 4934 patients) showed that both the presence of an MP component and the MP predominant subtype of lung ADC predicted worse DFS and OS after surgery. Lung ADC is divided into five major histological subtypes (lepidic, acinar, papillary, solid, and MP) according to the recommendation proposed by IASLC/ATS/ERS in 2011 [3]. These five major histological subtypes represent different aggressiveness characteristics [4]. Subsequent studies confirmed the predictive value of the new classification for prognosis and defined three subgroups: low-risk group (lepidic),intermediate-risk group (acinar and papillary) and high-risk group (solid and MP) [23–25]. Several studies in recent years have suggested that the MP predominant subtype predicts a poor prognosis for lung ADC [5–7]. Some studies agree on the adverse effects of the MP predominant subtype on DFS after lung ADC resection [9, 21, 22]. However, the effects of the MP predominant subtype on OS after lung ADC resection were not significantly different in the studies of Westaway et al. [20] and Sun et al. [21], although the HRs were>1. The reason for the lack of statistical significance might be that the sample number was relatively small, as there were only 8 and 22 MP-predominant subtype patients in the studies of Westaway et al. and Sun et al., respectively. Our results showed that in the MP predominant subgroup of 5 studies (including 271 patients with the MP predominant subtype), the pooled HRs of DFS and OS were 1.62 (95% CI 1.20–2.21) and 1.53 (95% CI 1.19–1.96), respectively.

In the subgroup with presence of an MP component, the pooled HRs of DFS and OS from 5 studies (including 311 MP-positive patients) were 1.80 (95% CI 1.14–2.85) and 2.26 (95% CI 1.46–3.52), respectively. Although the study of Yi et al. showed that the presence of an MP component could not predict the risk of recurrence after resection of lung ADC (HR 1.0, 95% CI 0.41–2.65, *P* = 0.919) [14], all the included patients were in stage I without lymph node involvement, and their conclusion might not be applicable to all patients with MP patterns. Recent studies showed that patients with an MP component had significantly higher rates of occult lymph node metastasis than patients without an MP component [10–12]. Two other studies confirmed that the presence of an MP component was closely related to lymph node metastasis [26, 27]. Combining these findings with the clear impact of TNM staging on prognosis after lung ADC resection further supported our results.

This is the first meta-analysis evaluating the impact of the MP pattern on the prognosis of lung ADC according to two subgroups: the MP component subgroup and the MP predominant subgroup. The heterogeneity was not obvious in all subgroup analyses (I^2^<50%, *P* >0.1). The summary data show that both patients with an MP component and patients with the MP predominant subtype have a higher probability of recurrence and worse overall survival. Current guidelines recommend adjuvant chemotherapy for patients whose TNM staging is higher than IIB after complete resection; stage IB and IIA patients with high risk factors may benefit from adjuvant chemotherapy after complete resection, especially patients with poorly differentiated tumours, vascular invasion, tumours with a diameter > 4 cm and visceral pleural invasion [28, 29]. According to the guidelines published by the Chinese Medical Association, adjuvant chemotherapy can also be considered for stage IB lung ADC patients whose pathological subtypes are solid or MP after complete resection [29]. Several recent studies have also reported that stage IB patients with MP subtype lung ADC can benefit from adjuvant chemotherapy [30–32]. Our meta-analysis results provide further evidence that lung ADC patients with an MP component may potentially benefit from adjuvant chemotherapy after complete surgical resection due to their higher relapse rates and poorer survival outcomes. However, this conclusion needs to be further confirmed in large prospective studies.

Our meta-analysis has some limitations: First, all the included studies were retrospective, and some of the research subjects’ biases were uncontrollable. Propensity score matching was not used to eliminate the influence of confounding factors on the observation results. Second, our meta-analysis only included some studies that defined the presence of an MP component as a proportion of the MP pattern > 5%. However, the prognosis of the part of lung ADC patients with MP pattern accounted for 1 to 5% after surgical resection has not been evaluated.

## Conclusion

Both the presence of an MP component and the MP predominant subtype are related to poor DFS and OS after lung ADC resection and represent adverse prognostic factors for lung ADC patients. However, there are some limitations in this meta-analysis, and quantitative stratification based on the proportion of the MP component is needed to explore its effect on the prognosis of lung ADC patients in the future.

## Supplementary information

**Additional file 1: Table S1.** The quality evaluation results of the included studies.

## Data Availability

The datasets generated and analyzed during the current study are available from the corresponding author on reasonable request.

## Abbreviations

MP: Micropapillary
ADC: Adenocarcinoma
DFS: Disease-free survival
OS: Overall survival
HR: Hazard ratio
CI: Confidence interval
IASLC/ATS/ERS: The International Association for the Study of Lung Cancer, the American Thoracic Society and the European Respiratory Society
STAS: Spread through air spaces
QUIPS: Quality In Prognosis Studies
TNM: Tumor node metastasis
SE: Standard error
