# Supplementary material for: Both the presence of a micropapillary component and the micropapillary predominant subtype predict poor prognosis after lung adenocarcinoma resection: a meta-analysis
Source: J Cardiothorac Surg. 2020 Jun 29;15:154. doi: 10.1186/s13019-020-01199-8 (PMC7325156; doi:10.1186/s13019-020-01199-8)
Supplement: Supplementary file 1 — Additional file 1: Table S1. The quality evaluation results of the included studies. [file 13019_2020_1199_MOESM1_ESM.docx]

Table S1. The quality evaluation results of the included studies

| **First Author(year)** | **Risk of Bias in Different Domains** | | | | | |
| --- | --- | --- | --- | --- | --- | --- |
|  | **Study Participation** | **Study Attrition** | **Prognostic Factor Measurement** | **Outcome Measurement** | **Study Confounding** | **Statistical Analysis and Reporting** |
| **Presence of micropapillary component** | | | | | | |
| Liu（2014）[17] | Low | Moderate | Moderate | Low | Low | Low |
| Tsubokawa（2016）[18] | Low | Low | Low | Low | Low | Low |
| Moon （2016）[19] | Low | Low | Low | Low | Low | Low |
| Yao（2016）[13] | High | Low | Moderate | Low | Low | Low |
| Yi（2018）[14] | Low | Low | Moderate | Low | Moderate | Low |
| **micropapillary predominant subtype** | | | | | | |
| Westaway（2013）[20] | Low | Moderate | Moderate | Low | Low | Low |
| Sun（2014）[21] | Low | Low | Low | Low | Low | Low |
| Warth （2015）[22] | Low | Low | Low | Low | Low | Low |
| Watanabe （2015）[8] | Moderate | Moderate | Low | Low | Low | Low |
| Zhang（2016）[9] | Low | Low | Moderate | Low | Low | Low |
